# Mucinous Carcinoma of the Breast With Neuroendocrine Differentiation: A Case Report of Cyto-Histopathology Findings

**DOI:** 10.7759/cureus.68015

**Published:** 2024-08-28

**Authors:** Anita B Sajjanar, Suhit Naseri, Pratibha Dawande, Sunita Vagha

**Affiliations:** 1 Department of Pathology, Datta Meghe Medical College, Datta Meghe Institute of Higher Education and Research, Nagpur, IND; 2 Department of Pathology, Jawaharlal Nehru Medical College, Datta Meghe Institute of Higher Education and Research, Wardha, IND

**Keywords:** immunohistochemistry staining, histopathological findings, cytological findings, rare invasive breast carcinoma, neuroendocrine differentiation, mucinous carcinoma

## Abstract

Mucinous breast carcinoma is a rare neoplasm. A minority of breast neoplasms exhibit a mucinous component, with purely mucinous cases being less frequent. It is more typically found in postmenopausal women. The etiology is multifactorial and involves dietary factors, reproductive factors, and hormonal factors. Mucinous carcinoma can grow to a large size at the time of diagnosis, although it typically grows slowly and palpable. Transcriptomic genetic studies have explained that mucinous tumors are of luminal A molecular subtype. Mucinous A tumors have different transcriptome characteristics than mucinous B tumors, which have a gene expression pattern resembling neuroendocrine (NE) carcinomas. Diagnosis of mucinous carcinoma with NE differentiation by fine needle aspiration cytology (FNAC) is reported infrequently. Histopathology is mandatory in the evaluation of mucinous breast carcinoma. NE carcinoma of the breast is an underestimated subtype of BC which has characteristics of heterogenicity, rarity, and poor differentiation. In this instance, we present a case of breast carcinoma exhibiting NE differentiation. A postmenopausal woman aged 63, with no family history of breast cancer, presented with a firm mass in the upper lateral quadrant of her right breast. This lump, causing discomfort for the past two years, was accompanied by nipple retraction and the discharge of bloody fluid. The clinical examination revealed the palpable presence of the lump. Ultrasonography-guided FNAC suggested Mucinous breast carcinoma with NE differentiation. The patient underwent a modified radical mastectomy, and the tissue was evaluated by immunohistochemistry which confirmed the diagnosis.

## Introduction

Mucinous breast carcinoma is a rare subtype, comprising only 2% of all breast cancers [1]. The morphological features resemble neuroendocrine (NE) tumors in the gastrointestinal tract and lung. It is commonly seen in postmenopausal women and has a favorable prognosis when diagnosed early [2]. The etiology is multifactorial and involves dietary factors, reproductive factors, and hormonal changes. Mucinous carcinoma can grow to a large size at the time of diagnosis, although it typically grows slowly and palpable [3]. Transcriptomic genetic studies have explained that mucinous tumors are of luminal A molecular subtype. Mucinous A tumors have different transcriptome characteristics than mucinous B tumors, which have a gene expression pattern resembling NE carcinomas [4]. Mammography often shows lobulated and circumscribed breast lesions, which are indicative of a mucinous carcinoma and on MRI manifest as a homogeneous strongly high signal intensity on T2-weighted images [5]. Mucinous cancer typically appears as a complicated mass with cystic and solid components, distal enhancement, microlobulation, and vascularity on ultrasonography [6]. Diagnosis of mucinous carcinoma with NE differentiation by fine needle aspiration cytology (FNAC) is reported infrequently [2]. Histopathology is mandatory in the evaluation of mucinous breast carcinoma. Feyrter and Hartmann described the NE differentiation of breast carcinoma in 1963, and it was adopted in classification by the World Health Organization (WHO) in 2003 and was modified in 2019 [7]. Breast cancer displaying NE differentiation is categorized into two groups according to the World Health Organization edition: Invasive carcinoma with more than 90% of cells exhibiting NE morphology be classified as neuroendocrine tumor or neuroendocrine carcinoma (NET or NEC), those with 10-90% of cells exhibiting NE morphology be classified as mixed invasive - not otherwise specified (NST) and NET/NEC, and those with less than 10% of cells exhibiting NE differentiation be classified as invasive carcinoma of NST, with an option for comment on the focal NE pattern [8]. NE breast carcinoma is observed in up to 20% of breast carcinomas, so it is difficult to evaluate because immunohistochemistry (IHC) for NE markers is rarely done. Hence, the actual incidence is difficult to assess [9].

## Case presentation

A postmenopausal woman aged 63 visited surgery OPD with swelling and pain in the right breast last two months. There was no similar family history. The clinical examination revealed a palpable lump in the upper lateral quadrant of her right breast accompanied by nipple retraction and the discharge of bloody fluid. There was no palpable mass in the left breast. Ultrasonography (USG) examination revealed a well-circumscribed mass with cystic and solid components. FNAC was advised. On USG-guided FNAC, the smears were highly cellular, comprising a monomorphic population arranged in discohesive sheets and occasional clusters. The tumor cells are plasmacytoid, having large eccentric nuclei, one to two prominent nucleoli, and salt-pepper chromatin with abundant cytoplasm. Smears were stained with hematoxylin & eosin, Papanicolaou, and Leishman. With these features, it was reported as mucinous carcinoma with NE differentiation. The patient underwent modified radical mastectomy, and the specimens were forwarded for histopathological examination. The gross findings revealed mastectomy specimen measured 12 x 9 cmx3.1 cm with elliptical skin and retracted nipple. The consistency of the lump varied from soft to hard, and there were irregular borders. The cut section showed a growth measuring 7.5 x 3x2 cm, which was gray-brown to gray-white, with a few cystic areas showing mucoid material, and a few hemorrhagic areas were also noted. Axillary lymph nodes were dissected with a size ranging from 0.5cm to 1.5cm. Diagnosis subsequently confirmed on histopathology along with all the surgical margins and lymph nodes were free from tumor deposits. Immunohistochemistry further confirmed the diagnosis with the tumor showing diffuse positivity for neuron-specific enolase, chromogranin, and synaptophysin. The tumor was positive for estrogen receptor and progesterone receptor and negative for human epidermal growth factor receptor (HER2/neu). Ki67 showed positive staining for <10%.

**Figure 1:**
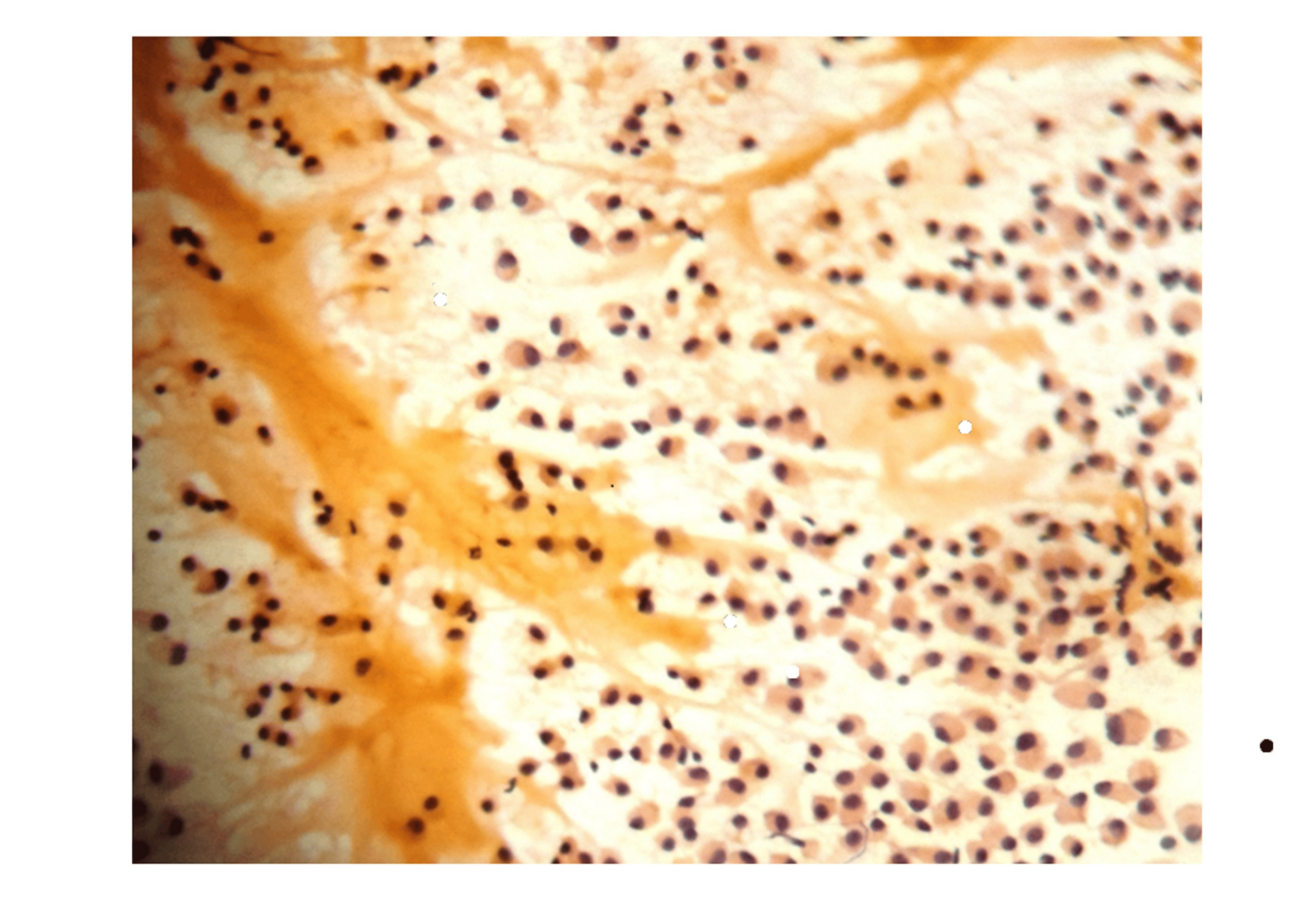
FNAC: plasmacytoid cells with chicken wire blood vessels (PAP, 10x) FNAC: Fine needle aspiration cytology

**Figure 2:**
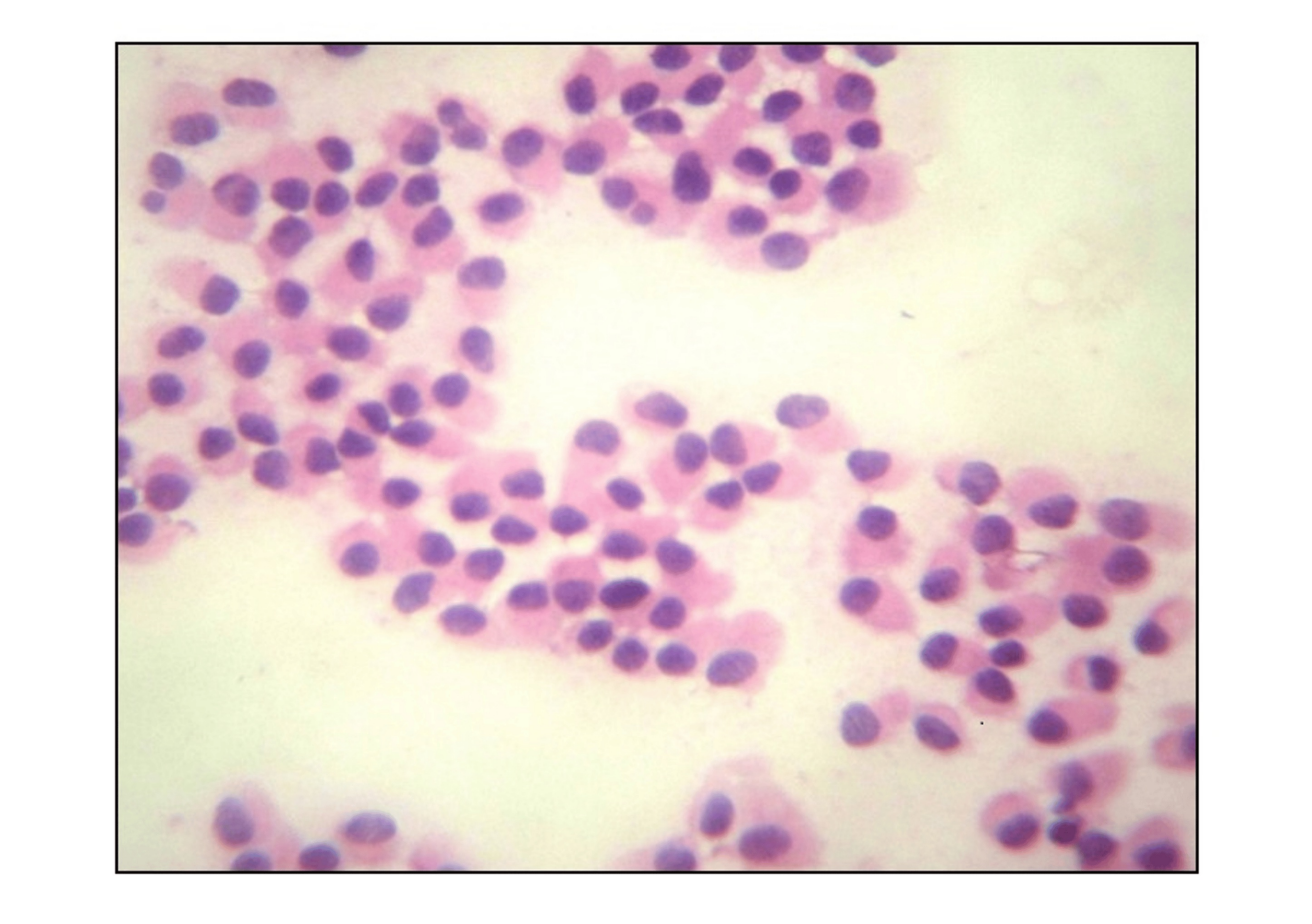
FNAC: plasmacytoid cells (H&E X40) FNAC: Fine needle aspiration cytology

**Figure 3:**
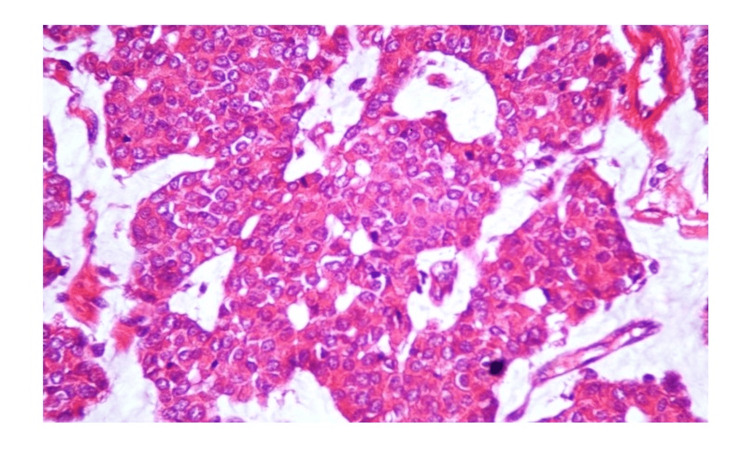
Histopathology findings: malignant cells arranged in nests (H&E, 40x)

**Figure 4:**
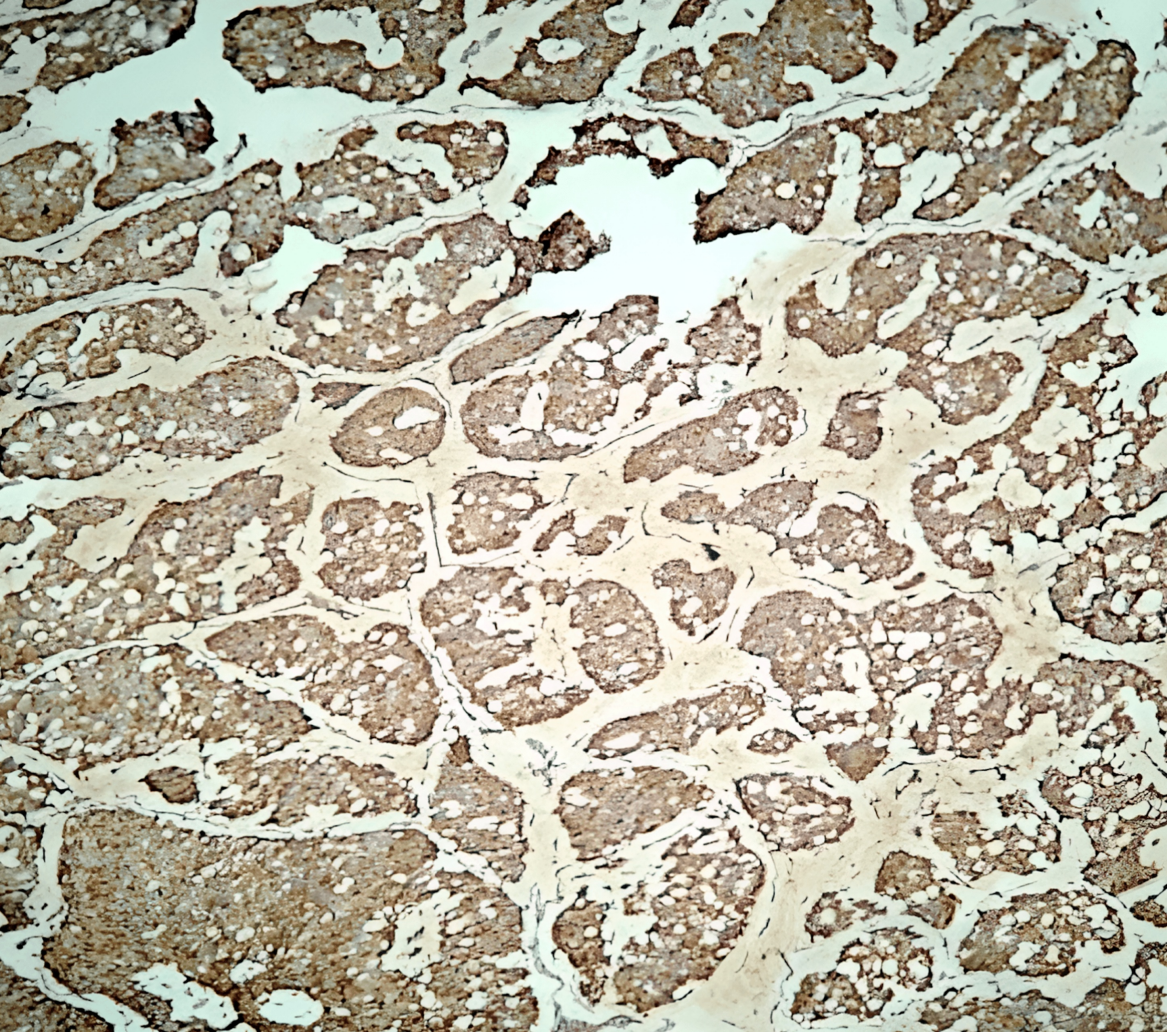
Tumor cells strongly positive for chromogranin (IHC,10x) IHC: Immunohistochemistry

**Figure 5:**
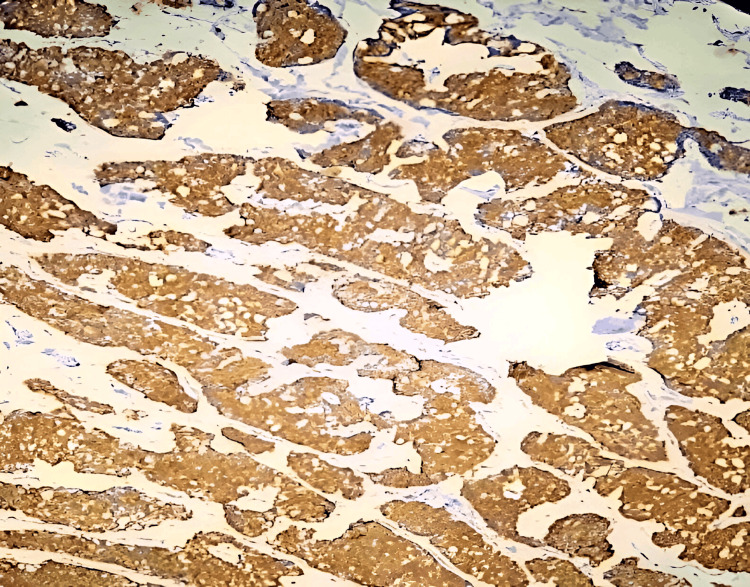
Tumor cells strongly positive for synaptophysin (IHC,10x).

**Figure 6:**
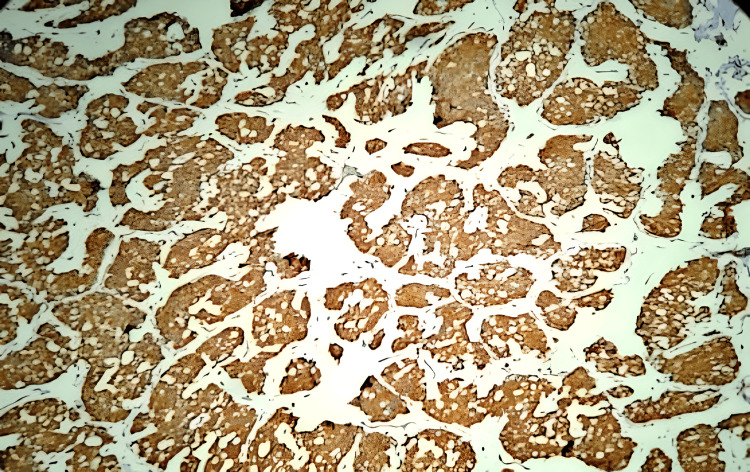
Tumor cells strongly positive for neuron-specific enolase (IHC, 10x)

**Figure 7:**
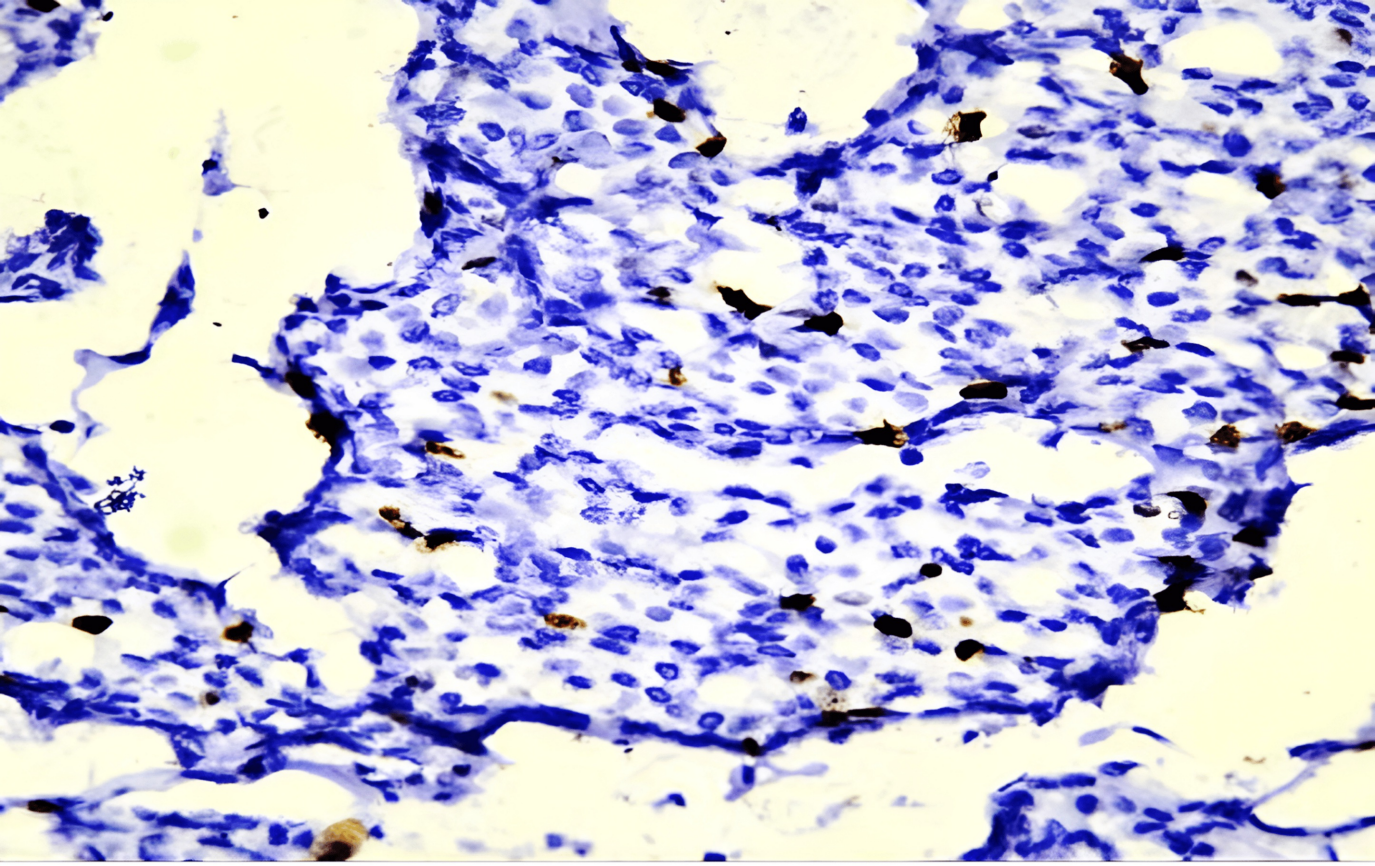
Tumor cells showing Ki67 proliferation index<10% (IHC,10x)

Taking into account the aforementioned observations, a conclusive diagnosis of breast mucinous carcinoma with NE differentiation was established (Figures 1-7). Later, the patient underwent chemotherapy and a follow-up up done for six months.

## Discussion

NE differentiation occurs in 2-5% of breast carcinomas, while pure NE carcinoma occurs in fewer than 0.1% of all breast cancers. It is a well-established kind of infiltrating ductal carcinoma that primarily affects older women and has a favorable prognosis [10]. Metastases are less common than other types of invasive malignancies. Mucinous breast carcinomas (mainly mixed type) are associated with lobular or ductal neoplasia (in situ or invasive) and some with focal NE differentiation [11]. The National Comprehensive Cancer Network (NCCN) Clinical Practice Guidelines in Oncology state that NE differentiation is commonly linked to mucinous carcinoma, with around 50% of tumors showing the presence of both NE differentiation and other identifiable markers. Mucinous carcinomas seldom have lymph node metastases [12]. The distinction between mucinous carcinoma and other mucinous lesions such as mucocele-like lesions, mucinous cysts, and mucinous spherulosis should be made. Mucinous malignancy is diagnosed cytologically based on the distinctive morphology and extensive extracellular mucin. Aspirate typically produces clear jelly-like mucus [13]. NE differentiation is an infrequent characteristic that sometimes emerges in breast carcinomas, displaying morphological patterns akin to NE malignancies found in various organs such as the esophagus, Meckel’s diverticulum, liver, pancreas, biliary tract, pelvic and otolaryngeal organs, as well as the breast [14]. Chromogranin A and synaptophysin are produced as specific products by NE cells. Despite the fact that NE differentiation has long been distinguished, its relevance remains uncertain. Because there is limited availability of long-term survival data, providing a prognosis for primary NE carcinoma of the breast proves challenging [15]. Previously, these tumors went by names such as argyrophilic breast carcinoma, breast carcinoid tumor, or endocrine carcinoma. However, contemporary recognition categorizes them as breast carcinomas displaying NE differentiation or primary NE breast carcinomas. Breast cancer in men has also been demonstrated to have NE differentiation. Clinically, there are no discernible or distinctive changes in presentation compared to various breast tumor types [16].

Sapino et al. documented the FNAC results of a series of primary NE breast carcinomas. The identification of intracytoplasmic granules played a crucial role in the detection of NE tumors by FNAC [17]. This case report highlights the usefulness of FNAC in correctly diagnosing mucinous carcinoma with NE differentiation and provides the earliest clue to diagnosis, later confirmed by histopathology and IHC. Although primary NE carcinoma of the breast is considered clinically aggressive and has a poor prognosis, early identification may result in a better prognosis [18].

## Conclusions

The exploration of NE carcinoma of the breast has not only deepened our understanding of its molecular intricacies but has also paved the way for innovative targeted molecular therapies by unraveling the distinctive molecular pathways and markers such as synaptophysin and chromogranin A. Given the limited availability of extended survival data, providing a prognosis for primary NE carcinoma of the breast proves challenging. Thus, FNAC is a beneficial, rapid, reliable, and cost-effective procedure for early and accurate diagnosis of these lesions with better prognosis. The chicken wire blood vessels and plasmacytoid cells with a nucleus with salt and pepper chromatin give a clue to the diagnosis.
